# **Synbiotic containing** ***Bacillus coagulans*** **and fructo-oligosaccharides for functional abdominal pain in children**

**Published:** 2015

**Authors:** Hossein Saneian, Zahra Pourmoghaddas, Hamidreza Roohafza, Ali Gholamrezaei

**Affiliations:** 1 *Child Growth and Development Research Center, Isfahan University of Medical Sciences, Isfahan, Iran*; 2 *Psychosomatic Research Center, Isfahan University of Medical Sciences, Isfahan, Iran*; 3 *Poursina Hakim Research Institute, Isfahan, Iran*

**Keywords:** Abdominal pain, Child, Probiotic, Prebiotic, Synbiotic

## Abstract

**Aim**: We evaluated the effectiveness of a synbiotic in the treatment of childhood functional abdominal pain (FAP).

**Background**: Probiotics are effective in the treatment of functional gastrointestinal disorders in adult patients, but there is lack of information in children.

**Patients and methods**: Children with FAP, based on the Rome III criteria (*n* = 115, aged 6-18 years), were randomized to receive either synbiotic (*Bacillus coagulans, *Unique IS-2, 150 million spore plus FOS, 100 mg) twice daily or placebo for four weeks. Treatment response was defined as ≥ 2-point reduction in the 6-point self-rated pain scale or “no pain”. Physician-rated global severity and improvement were also evaluated. Patients were followed for a total of 12 weeks.

**Results**: Eighty-eight patients completed the trial (45 with synbiotic). Response rate was higher with synbiotic than placebo after medication (60% vs. 39.5%, P = 0.044), but was not different between the two groups at week 12 (64.4% vs. 53.4%, P = 0.204). Difference between the two groups regarding the physician-rated global severity over the study period was not statistically significant (z = -1.87, P = 0.062). There was no significant difference between the two groups in physician-rated global improvement (week 4, P = 0.437; week 12, P = 0.111). Receiving synbiotic (OR 2.608, 95% CI: 1.01-6.68) and baseline pain score (OR 2.21, 95% CI: 1.19-4.10) were predictors of treatment response after medication.

**Conclusion**: The synbiotic containing *Bacillus coagulans* and FOS seems to be effective in the treatment of childhood FAP. Further trials are recommended in this regard.

## Introduction

Functional abdominal pain (FAP) is a common functional gastrointestinal disorder (FGID) among school-aged children with the prevalence of about 8% in western counties (1). It is associated with worse quality of life, increased absenteeism of the child from school and the parents from work (2), and substantial healthcare utilization (3). Accordingly, FAP in children is a significant burden on the healthcare system and the society.

The pathophysiology of FAP as well as other pain-related FGIDs is not yet clear. Abnormal gastrointestinal motility (4), visceral hypersensitivity (5), altered brain-gut interaction (6), low-grade inflammation (7), psychosocial disturbance (8), and the gastrointestinal microbiota (9) are proposed contributors in the development of FGIDs. Since the pathophysiology of these disorders is not completely understood, treatment of FAP in children remains a challenge for clinicians. Various pharmacological and non-pharmacological therapies are studied up to now, but most of them failed to provide substantial therapeutic effects (10). 

Studies have shown altered gastrointestinal microbiota in adult and pediatric patients with FGIDs (9). Abnormal gastrointestinal microbiota may not only affect intestinal immune function and epithelial permeability (11,12), but also may have effect on the enteric as well as central nervous system and the brain-gut interaction contributing to the development of FGIDs (7,13-17). It is not yet clear if the observed abnormal gastrointestinal microbiota in patients with FGIDs is a cause or a consequence of other pathophysiological mechanisms such as gastrointestinal motility and secretion (9). Nevertheless, the microbiota has been a therapeutic target by dietary manipulation, non-absorbable antibiotics, prebiotics, probiotics, and synbiotics (9).

Probiotics are live microorganisms with health benefits on the host when consumed in adequate amounts. Prebiotics are non-digestible food ingredients that selectively stimulate the growth of beneficial bacteria, which are already present in the host (e.g. bifidobacteria). A combination of prebiotic and probiotic is called synbiotic (18). Growing number of studies have evaluated the efficacy of probiotics in the treatment of various gastrointestinal conditions (19). There are evidence supporting the effects of probiotics on some of the above-mentioned underlying mechanisms of FGIDs including gastrointestinal sensitivity, motility, immune function, permeability, and microbiota (9). Recent meta-analyses have shown that probiotics are effective in the treatment of pain-related FGIDs mainly irritable bowel syndrome (IBS) in adult and pediatric patients (20-23). However, there are few well-designed studies on the efficacy of prebiotics and synbiotics in this regard (9). Also, only few trials have evaluated the effectiveness of probiotics in the treatment of pain-related FGIDs in children, which have mainly focused on IBS and had limited number of FAP patients (21). According to the lack of evidence in pediatric patients, we conducted a randomized, placebo-controlled trial on the efficacy of a synbiotic containing *Bacillus coagulans* plus fructo-oligosaccharides (FOS) in the treatment of FAP in children. 

## Patients and Methods

**Study participants**

A clinical trial was conducted from February through December 2013 at an outpatient tertiary clinic of pediatric gastroenterology in the city of Isfahan, Iran. Eligible participants were children in the age range of 6 to 18 years who fulfilled the Rome III diagnostic criteria for FAP. The criteria include episodic/continuous abdominal pain at least once per week for at least two months (24). Children with alarm signs (e.g. anemia, rectal bleeding, etc.) were further evaluated for organic diseases. None of the included children fulfilled the Rome III criteria for IBS or functional dyspepsia. Those with organic diseases as the cause of abdominal pain, other concomitant gastrointestinal disorders, or immune-compromised conditions, and those with recent history (preceding two months) of or current treatment with antibiotics, antidepressants, antispasmodics, or probiotics were not included into the study. The ethics Committee of the Isfahan University of Medical Sciences approved the study and informed consent was obtained from the parents. 

**Study design and sample size**

The study was designed as a randomized, double-blind, placebo-controlled trial. Using random numbers in four blocks (generated by software), synbiotic and placebo containing drug bottles were coded by a pharmacist. Allocation was concealed and the attending physician, patients, and outcome assessor were unaware of the drug codes until the end of the study. Estimating treatment response of 70% for probiotic and 40% for placebo (25), and at a power of 80% and a significance level of 0.05, we needed 41 children per group. The trial was registered in the Australian New Zealand clinical trial registry (ACTRN12613000158763).

**Intervention**

The synbiotic group received synbiotic tablets twice daily (Lactol®, Bioplus Life Sciences Pvt., Ltd., Bangalore, India) for a duration of four weeks. This synbiotic is composed of the probiotic *Bacillus Coagulans* (Unique IS-2, 150 million spores) plus FOS (100 mg). The placebo group received placebo tablets in a same order. Treatment adherence was examined after two weeks of medication by telephone interview and also at the 4-week visit by counting the remained pills. 

**Outcome measures and follow-up**

A single trained interviewer who was blinded to the allocation sequence and study arms interviewed with children and parents. The primary outcome measure was treatment response defined as at least 2 point reduction in the Wong-Baker FACES® Pain Rating Scale or “no pain” after medication. This pain rating scale is a well-known instrument for measuring pain intensity in children by self-report. Consisting of six faces that show pain effect, the scale ranges from a relaxed face on the left (no hurt scored 0) to a face showing intense pain on the right (hurts worse scored 5). The child was asked to choose the face he/she has at the time of pain (26).

**Figure 1:**
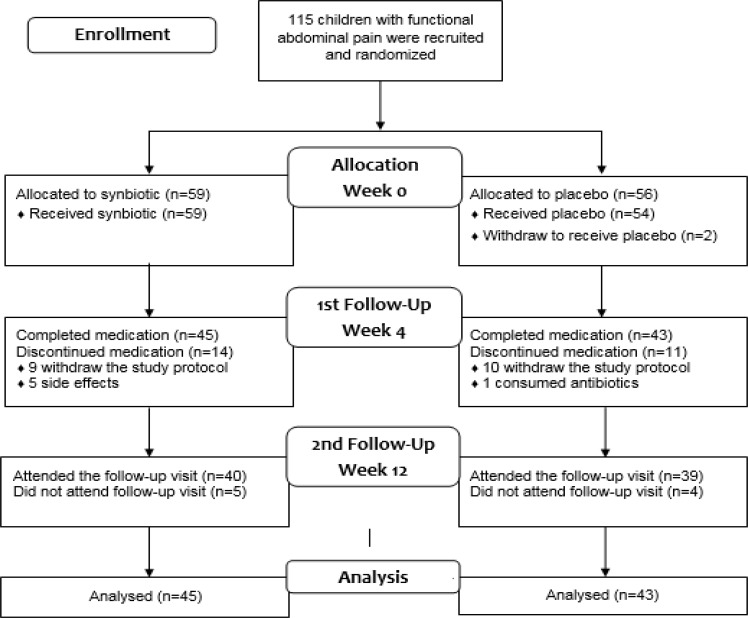
Patients’ flow diagram

Secondary outcomes during the 4-week medication included the physician-rated global severity and improvement using the Clinical Global Impression Severity and Improvement scales (CGI-S, CGI-I). The CGI-S and CGI-I are brief 7-point physician-rated scales of the global severity of the illness and improvement by the treatment, respectively. The severity is scored from 1 (Normal) to 7 (among the most extremely ill patients) and the improvement is scored from 1 (very much improved) to 7 (very much worse) (27). Adverse events were assessed after two weeks of medication by telephone interview and also at the 4-week visit using a checklist including side effects of probiotics. In case of severe side effects, drug was discontinued. To test durability of the response to medication, primary and secondary outcomes’ measurements were repeated 8 weeks after drugs discontinuation (the 12-week follow-up visit) (figure 1). 

**Statistical analysis**

Data are presented as mean ± SD or number (percent). Data were assessed for a normal distribution before analyses using Kolmogorov-Smirnov test. Between-group comparisons were done with independent t-test and chi-square test. Equivalent non-parametric tests were applied when appropriate. For these tests, the Statistical Package for Social Sciences software version 16.0 (SPSS Inc., Chicago, IL, USA) was used. We compared the two groups regarding the study outcomes based on the intention-to-treat principle. We used random intercept linear mixed model with compound symmetric covariance structure for the analysis of the effects of time and group on the changes of pain and CGI-S scores using the STATA software (Stata/IC 9.2, StataCorp LP, TX, USA). Models were adjusted for age, sex, and baseline value of each outcome variable. Also, logistic regression models were conducted to find predictors of treatment response. A two-sided p-value of less than 0.05 was considered statistically significance in all analyses.

## Results

During the study period, 115 children with FAP were assigned to either the synbiotic (n= 59) or the placebo (n= 56) groups. Twenty-one patients (9 in the probiotic and 12 in the placebo group) withdraw the study after assignment not related to side effects. Five patients from the synbiotic group discontinued medication due to side effects; two had worsened pain, one loss of appetite, one diarrhea, and one fever. In the placebo group, one patient used antibiotics during the medication period. A total of 88 patients completed the 4-week medication period. Nine patients (5 in the probiotic and 4 in the placebo group) did not attend the follow-up visit at week 12 (patients’ flow diagram). There was no difference between patients who withdraw the study with those who remained in the study regarding demographic factors or baseline values of the study outcome variables, except higher CGI-S score in those who remained in the study (5.3 ± 1.0 vs. 4.3 ± 1.2, p< 0.001). 

**Baseline characteristics of the patients**

Demographic data and baseline characteristics are presented in table 1. Mean age of the total participants was 8.5 ± 2.1 years and 48 (55.1%) were female. There was no significant difference between the two groups in demographic data or baseline characteristics.

**Table 1:** Comparison of demographic data and baseline characteristics between the two groups

	Synbiotic (n= 45)	Placebo (n= 43)	P-value
Age, year	9.0 ± 2.2	8.5 ± 2.2	0.384*
Boy/Girl	25/20	24/19	0.538†
Father education			
0-5 year	12 (26.6)	7 (16.2)	0.474‡
6-12 year	22 (48.8)	24 (55.8)	
>12 year	11 (24.4)	12 (27.9)	
Family income a			
Low	6 (13.3)	9 (20.9)	0.106‡
Middle	24 (53.3)	26 (60.4)	
High	15 (33.3)	8 (18.6)	
Pain score	3.6±0.9	3.6±0.8	0.608‡
CGI-S	5.2±1.1	5.4±0.9	0.849‡

Data are presented as mean ± SD or number (%). CGI-S: Clinical Global Impression Severity Scale,

* Independent Sample t-Test,

† Chi-square Test,

‡ Mann-Whitney U Test,

a Based on the Iranian Rial Currency

**Table 2:** Comparison of primary and secondary outcomes between the two groups

	Synbiotic n = 45	Placebo n = 43	P-value
Change in pain score			
Week 4	-1.7 ± 1.5	-1.6 ± 1.5	0.543*
Week 12	-2.1 ± 1.4	-1.8 ± 1.4	0.335 *
Change in CGI-S score			
Week 4	-3.3 ± 1.4	-3.0 ± 1.7	0.463 *
Week 12	-3.5 ± 1.4	-3.1 ± 1.5	0.160 *
CGI-I score at week 4	2.2 ± 1.2	2.5 ± 1.4	0.437 *
CGI-I score at week 12	1.9 ± 1.1	2.4 ± 1.4	0.111 *
Response rate at week 4	27 (60)	17 (39.5)	0.044†
Response rate at week 12	29 (64.4)	23 (53.4)	0.204†

Data are presented as mean ± SD. CGI-S, CGI-I: Clinical Global Impression Scale-Severity, -Improvement,

* Mann-Whitney U Test,

† Chi-square Test

**Primary and secondary outcome measures**

Univariate comparisons of the primary and secondary outcomes between the two groups are presented in table 2. Treatment response rate in the synbiotic and placebo groups was 60% and 39.5% at week 4 (p= 0.044) and 64.4% and 53.4% at week 12 (p= 0.204), respectively. There was no significant difference between the two groups in change of the CGI-S score after 4 weeks (p= 0.463) or after 12 weeks (p= 0.160). Also, there was no significant difference between the two groups in the CGI-I score at week 4 (p= 0.437) or at week 12 (p= 0.111). 

**Figure 2:**
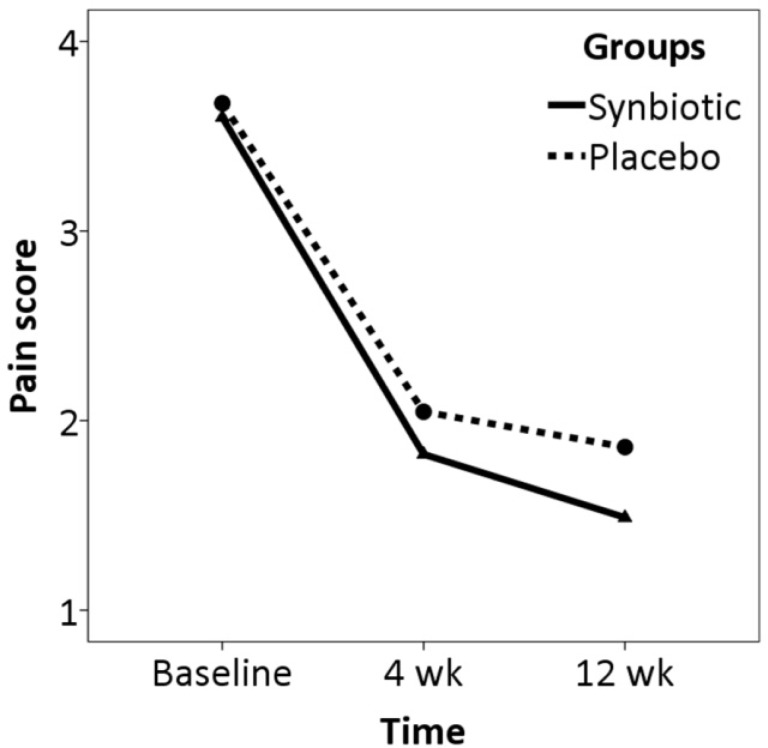
Trend of change in pain score over the study period

**Figure 3:**
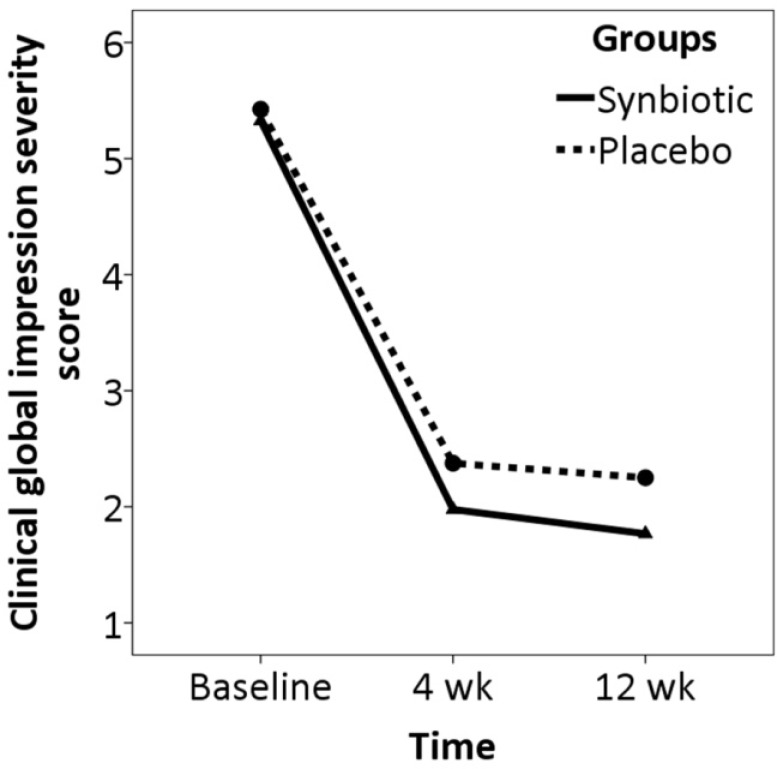
Trend of change in clinical global impression severity score over the study period

Intention-to-treat analysis controlling for demographic data and baseline characteristics showed no significant difference between the two groups in trend of changes in pain severity over the study period (z= -1.09, p= 0.276) (figure 2). A non-significant difference was found between the two groups in trend of changes in CGI-S over the study period (z= -1.87, p= 0.062) (figure 3). 

**Predictors of treatment response**

Results of the logistic regression analyses examining predictors of treatment response at weeks 4 and 12 are summarized in table 3. Receiving synbiotic (OR=2.608, 95% CI: 1.017-6.687) and baseline pain score (OR= 2.213, 95% CI: 1.192-4.109) were associated with treatment response after medication. At week 12, only baseline pain score remained significantly associated with treatment response (OR= 1.868, 95% CI: 1.027-3.398). 

**Treatment adherence and side effects**

Patients in the synbiotic and placebo group consumed 82.9 ± 17.2% and 89.4 ± 10.6% of the drugs, respectively. Comparison of side effects between the two groups is summarized in table 4. The synbiotic group experienced more dry mouth than the placebo group during medication (44.4% vs. 23.2%, p= 0.024). Other possible side effects were comparable between the two groups.

**Table 3:** Logistic regression analysis examining predictors of response at weeks 4 and 12

	Response at week 4		Response at week 12	
	OR (95% CI)	P-value	OR (95% CI)	P-value
Synbiotic vs. Placebo	2.60 (1.01-6.68)	0.046	1.88 (0.74-4.79)	0.184
Age	0.98 (0.79-1.21)	0.869	0.91 (0.73-1.12)	0.385
Girl vs. Boy	0.93 (0.34-2.48)	0.887	0.80 (0.30-2.12)	0.658
Parent education	0.90 (0.43-1.88)	0.794	1.17 (0.56-2.41)	0.670
Family income	1.10 (0.48-2.51)	0.821	0.92 (0.41-2.06)	0.855
Baseline pain	2.21 (1.19-4.10)	0.012	1.86 (1.02-3.39)	0.041
Nagelkerke R Square	0.173		0.119	

**Table 4:** Comparison of side effects between the two groups

	Synbiotic (n= 45)	Placebo (n= 43)	*P*-value
Insomnia	4 (8.8)	1 (2.3)	0.202
Nausea	1 (2.2)	1 (2.3)	0.747
Drowsiness	11 (24.4)	7 (16.2)	0.247
Dry mouth	20 (44.4)	10 (23.2)	0.024
Diarrhea	1 (2.2)	0	0.517
Vomiting	1 (2.2)	0	0.517
Fatigue	2 (4.4)	6 (13.9)	0.112
Headache	3 (6.6)	1 (2.3)	0.335
Dizziness	4 (8.8)	2 (4.6)	0.372
Allergic reaction	0	0	-
Loss of appetite	8 (17.7)	8 (18.6)	0.548

Data are presented as number (%)

## Discussion

Probiotics are increasingly being studied in the treatment of various gastrointestinal disorders considering their effects on the gastrointestinal immune and sensory functions as well as microbiota (19). Several placebo-controlled trials have shown the effectiveness of probiotics in the treatment of pain-related FGIDs, mainly IBS, in adult patients (20-23). However, few studies are done in pediatric patients. In this regard, Gawrońska and colleagues in a placebo-controlled trial determined the efficacy of 4-week treatment with *Lactobacillus rhamnosus *GG (LGG, 3 × 109 CFU, twice daily) for 104 children with IBS, FAP, or functional dyspepsia. Authors found a higher rate of treatment success (defined as no pain in the FACE pain scale) and improvement (defined as change by at least two faces scores) in those who received LGG compared with the placebo group (73% vs. 53.6%). However, sub-group analysis showed significant difference in treatment success of probiotic over placebo only in IBS patients (33.3% vs. 5.3%) (25). In other placebo-controlled trial, Francavilla and colleagues determined the efficacy of 8-week treatment with LGG (3 × 109 CFU, twice daily) in 83 children with IBS and 58 children with FAP. Authors found a significant reduction in the severity and frequency of pain in IBS patients (28). These investigators also evaluated intestinal permeability and found a decrease in abnormal permeability test by the probiotic only in IBS patients (28). In contrast to these studies, Bausserman and Michail found no benefits for 6-week treatment with LGG (1010 CFU, twice daily) over placebo in their study including 50 children with IBS, except a decrease in abdominal distension (29). A recent meta-analysis by Horvath and colleagues on the above-mentioned studies showed that LGG is effective in treatment of overall pediatric population with pain-related FGIDs, particularly IBS. However, no significant effectiveness was observed for children with FAP or functional dyspepsia (21). It must be noted that these studies have mainly focused on IBS and had limited number of patients with FAP.

Regarding children with FAP, there is only one available report with focus on this patient population. The study by Romano and colleagues in a sample of children with FAP showed an improvement of pain intensity with 4-week treatment of *Lactobacillus reuteri* (108 CFU twice day) over placebo 30. We examined the efficacy of 4-week treatment with a synbiotic containing *Bacillus coagulans* and FOS in a relatively large sample of children with FAP. We found a higher treatment response rate with synbiotic over placebo after medication (60% vs. 40%) which was also independent from demographic and baseline characteristics of the patients. Although no deterioration was observed over the 8-week follow-up period, the beneficial effect of synbiotic did not remain significant over placebo 8 weeks after drug discontinuation. In contrast to these results, the study by Francavilla and colleagues showed that improvement of IBS symptoms by probiotic is sustained for 8 weeks follow-up after drug discontinuation. Treatment duration was 8 weeks in the mentioned study (compared to 4 weeks in our study) and the study focused on IBS patients (28). 

Several factors may contribute to the efficacy of probiotics in treatment of FGIDs. Although there is no real head to head comparative study on different probiotics, there is evidence that some species (e.g. bifidobacteria) may be more effective than others in the treatment of pain-related FGIDs (31). Also, there is assumption that multistrain probiotic mixture may be more effective than single strain products 32. In this regard, Guandalini and colleagues investigated the efficacy of a 6-week treatment with a multistrain probiotic mixture comprising 8 different strains of lactic acid bacteria in 59 children with IBS. Authors found that the probiotic is superior to placebo in improving abdominal pain and bloating 33. Studies in adult patients also showed the same results (31). However, a recent meta-analysis on various gastrointestinal diseases did not show difference between the efficacies of single or multiple species probiotics (34). In this regard, head to head studies are warranted.

To have beneficial effects for the host, probiotics should be taken in adequate dosage and duration. Treatment duration was 4 weeks in our study in accordance with the Rome committee recommendation in design of treatment trials on FGIDs (35). In previous studies that examined probiotics for the treatment of chronic gastrointestinal problems treatment duration was at least 4 weeks (36). Regarding pain-related FGIDs, treatment duration varied from 4 to 12 weeks in adult and pediatric patients (20,21,31). The heterogeneity between previous studies regarding treatment dose and duration may contribute to differences in the reported results. A recent consensus from the European Society for Primary Care Gastroenterology recommended that probiotic should be taken for at least one month with dosage based on the available evidence and manufacturers’ recommendations (36). A dose response effect is reported for the efficacy of probiotic in IBS treatment (37). However, recent meta-analysis by Ritchie and Romanuk on the efficacy of probiotics for gastrointestinal diseases found some minor influence for treatment dosage and no significant effect of duration on therapeutic efficacy (34). The optimal dose and treatment duration of probiotics are not clearly established and further trials are required in this regard.

Probiotics are generally safe in children (38). We found no severe adverse effects with *Bacillus coagulans* in children aged 6-18 years. Five patients (8%) had to stop medication due to side effects. Other observed side effects were comparable between the synbiotic and placebo groups. Previous studies in pediatric patients with chronic gastrointestinal disorders showed that probiotics are well tolerated with no adverse effects (39,40). 

There are some limitations to our study. The placebo response in our study was unexpectedly high and a larger sample of patients was required. Also, as recommended by the Rome committee, at least 6 months follow-up is required to establish long-term efficacy of the treatment for FGIDs (35). While there were limited reports on the effectiveness of probiotics on childhood FAP, it was reasonable to follow patients for a shorter duration in our study. However, according to the results of this study, further trials with longer follow-ups are warranted. 

The synbiotic containing *Bacillus coagulans* and FOS seems to be effective in the treatment of childhood FAP. It is generally well tolerated with no severe adverse effect. A 4-week treatment with this synbiotic, however, does not have long-term effects. Further trials with longer treatment and follow-up duration in a larger sample of children with FAP are recommended.

## References

[1] Chitkara DK, Rawat DJ, Talley NJ (2005). The epidemiology of childhood recurrent abdominal pain in Western countries: a systematic review. Am J Gastroenterol.

[2] Saps M, Seshadri R, Sztainberg M, Schaffer G, Marshall BM, Di Lorenzo C (2009). A prospective school-based study of abdominal pain and other common somatic complaints in children. J Pediatr.

[3] Dhroove G, Chogle A, Saps M (2010). A million-dollar work-up for abdominal pain: is it worth it?. J Pediatr Gastroenterol Nutr.

[4] Kellow JE, Delvaux M, Azpiroz F, Camilleri M, Quigley EM, Thompson DG (1999). Principles of applied neurogastroenterology: physiology/motility-sensation. Gut.

[5] Van Ginkel R, Voskuijl WP, Benninga MA, Taminiau JA, Boeckxstaens GE (2001). Alterations in rectal sensitivity and motility in childhood irritable bowel syndrome. Gastroenterology.

[6] Mayer EA, Tillisch K (2011). The brain-gut axis in abdominal pain syndromes. Annu Rev Med.

[7] Hughes PA, Zola H, Penttila IA, Blackshaw LA, Andrews JM, Krumbiegel D (2013). Immune activation in irritable bowel syndrome: can neuroimmune interactions explain symptoms?. Am J Gastroenterol.

[8] Levy RL, Olden KW, Naliboff BD, Bradley LA, Francisconi C, Drossman DA (2006). Psychosocial aspects of the functional gastrointestinal disorders. Gastroenterology.

[9] Simren M, Barbara G, Flint HJ, Spiegel BM, Spiller RC, Vanner S (2013). Intestinal microbiota in functional bowel disorders: a Rome foundation report. Gut.

[10] Di Lorenzo C, Colletti RB, Lehmann HP, Boyle JT, Gerson WT, Hyams JS (2005). Chronic Abdominal Pain in Children: a Technical Report of the American Academy of Pediatrics and the North American Society for Pediatric Gastroenterology, Hepatology and Nutrition. J Pediatr Gastroenterol Nutr.

[11] Barbara G, Zecchi L, Barbaro R, Cremon C, Bellacosa L, Marcellini M (2012). Mucosal permeability and immune activation as potential therapeutic targets of probiotics in irritable bowel syndrome. J Clin Gastroenterol.

[12] Barbara G, Stanghellini V, Cremon C, De Giorgio R, Gargano L, Cogliandro R (2008). Probiotics and irritable bowel syndrome: rationale and clinical evidence for their use. J Clin Gastroenterol.

[13] Cryan JF, Dinan TG (2012). Mind-altering microorganisms: the impact of the gut microbiota on brain and behaviour. Nat Rev Neurosci.

[14] Collins SM, Surette M, Bercik P (2012). The interplay between the intestinal microbiota and the brain. Nat Rev Microbiol.

[15] Foster JA, Vey Neufeld KA (2013). Gut-brain axis: how the microbiome influences anxiety and depression. Trends Neurosci.

[16] Dinan TG, Cryan JF (2013). Melancholic microbes: a link between gut microbiota and depression?. Neurogastroenterol Motil.

[17] Wang Y, Kasper LH (2014). The role of microbiome in central nervous system disorders. Brain Behav Immun.

[18] Schrezenmeir J, de VM (2001). Probiotics, prebiotics, and synbiotics--approaching a definition. Am J Clin Nutr.

[19] Sanders ME, Guarner F, Guerrant R, Holt PR, Quigley EM, Sartor RB (2013). An update on the use and investigation of probiotics in health and disease. Gut.

[20] Hoveyda N, Heneghan C, Mahtani KR, Perera R, Roberts N, Glasziou P (2009). A systematic review and meta-analysis: probiotics in the treatment of irritable bowel syndrome. BMC Gastroenterol.

[21] Horvath A, Dziechciarz P, Szajewska H (2011). Meta-analysis: Lactobacillus rhamnosus GG for abdominal pain-related functional gastrointestinal disorders in childhood. Aliment Pharmacol Ther.

[22] Moayyedi P, Ford AC, Talley NJ, Cremonini F, Foxx-Orenstein AE, Brandt LJ (2010). The efficacy of probiotics in the treatment of irritable bowel syndrome: a systematic review. Gut.

[23] McFarland LV, Dublin S (2008). Meta-analysis of probiotics for the treatment of irritable bowel syndrome. World J Gastroenterol.

[24] Rasquin A, Di Lorenzo C, Forbes D, Guiraldes E, Hyams JS, Staiano A (2006). Childhood functional gastrointestinal disorders: child/adolescent. Gastroenterology.

[25] Gawronska A, Dziechciarz P, Horvath A, Szajewska H (2007). A randomized double-blind placebo-controlled trial of Lactobacillus GG for abdominal pain disorders in children. Aliment Pharmacol Ther.

[26] Stinson JN, Kavanagh T, Yamada J, Gill N, Stevens B (2006). Systematic review of the psychometric properties, interpretability and feasibility of self-report pain intensity measures for use in clinical trials in children and adolescents. Pain.

[27] National Institute of Mental Health (1985). Rating scales and assessment instruments for use in pediatric psychopharmacology research. Psychopharmacol Bull.

[28] Francavilla R, Miniello V, Magista AM, De Canio A, Bucci N, Gagliardi F (2010). A randomized controlled trial of Lactobacillus GG in children with functional abdominal pain. Pediatrics.

[29] Bausserman M, Michail S (2005). The use of Lactobacillus GG in irritable bowel syndrome in children: a double-blind randomized control trial. J Pediatr.

[30] Romano C, Ferrau' V, Cavataio F, Iacono G, Spina M, Lionetti E (2014). Lactobacillus reuteri in children with functional abdominal pain (FAP). J Paediatr Child Health.

[31] Brenner DM, Moeller MJ, Chey WD, Schoenfeld PS (2009). The utility of probiotics in the treatment of irritable bowel syndrome: a systematic review. Am J Gastroenterol.

[32] Chapman CM, Gibson GR, Rowland I (2011). Health benefits of probiotics: are mixtures more effective than single strains?. Eur J Nutr.

[33] Guandalini S, Magazzu G, Chiaro A, La Balestra V, Di Nardo G, Gopalan S (2010). VSL#3 improves symptoms in children with irritable bowel syndrome: a multicenter, randomized, placebo-controlled, double-blind, crossover study. J Pediatr Gastroenterol Nutr.

[34] Ritchie ML, Romanuk TN (2012). A meta-analysis of probiotic efficacy for gastrointestinal diseases. PLoS One.

[35] Irvine EJ, Whitehead WE, Chey WD, Matsueda K, Shaw M, Talley NJ (2006). Design of treatment trials for functional gastrointestinal disorders. Gastroenterology.

[36] Hungin AP, Mulligan C, Pot B, Whorwell P, Agreus L, Fracasso P (2013). Systematic review: probiotics in the management of lower gastrointestinal symptoms in clinical practice -- an evidence-based international guide. Aliment Pharmacol Ther.

[37] Whorwell PJ, Altringer L, Morel J, Bond Y, Charbonneau D, O'Mahony L (2006). Efficacy of an encapsulated probiotic Bifidobacterium infantis 35624 in women with irritable bowel syndrome. Am J Gastroenterol.

[38] Michail S, Sylvester F, Fuchs G, Issenman R (2006). Clinical efficacy of probiotics: review of the evidence with focus on children. J Pediatr Gastroenterol Nutr.

[39] Szajewska H, Skorka A, Ruszczynski M, Gieruszczak-Bialek D (2013). Meta-analysis: Lactobacillus GG for treating acute gastroenteritis in children--updated analysis of randomised controlled trials. Aliment Pharmacol Ther.

[40] Bernaola AG, BadaMancilla CA, Carreazo NY, Rojas Galarza RA (2013). Probiotics for treating persistent diarrhoea in children. Cochrane Database Syst Rev.

